# Editorial: The Future of the Leader-Member Exchange Theory

**DOI:** 10.3389/fpsyg.2021.736710

**Published:** 2021-08-19

**Authors:** Erich C. Fein, Aharon Tziner

**Affiliations:** ^1^School of Psychology and Counselling and Centre for Health Research, University of Southern Queensland, Toowoomba, QLD, Australia; ^2^Schools of Business Administration, Netanya Academic College and Peres Academic Center, Netanya, Israel

**Keywords:** leader-member-exchange, leadership patterns, antecedents of LMX, outcomes of LMX, LMX as moderator or mediator variable

In considering the Frontiers Research Topic “The Future of Leader-Member Exchange Theory” we present a retrospective overview of the key topics and organizing themes across the multiple articles within this special issue of Frontiers in Psychology.

There is no doubt we live in trying times because of effects related to the lingering COVID-19 pandemic. Accordingly, the difficulties related to life at work, both in traditional offices and when working remotely and online, has increased the importance of organizational leaders in mitigating the effects of dysfunctional workplace environments, and in compensating for incomplete or developing workplace systems. In addition, we find the workforce in today's world more diverse in terms of culture and respective value orientations, personality traits, and other individual differences. However, less is known about the effects of individuals' dispositional differences on LMX (e.g., Maslyn et al., 2017). In addition, even less is known about the effects of cultural and demographic parameters on leader–member interrelations, and their impact on job performance. We expect that such diversity will only increase as the continuing effects of COVID-19 change national and international economies, and the composition of the workforce, in unexpected ways.

One proposition underlying leader-member exchange (LMX) theory is that managers tend to employ different management styles for each of their subordinates [Graen and Uhl-Bien, 1995; see also Waismel-Manor et al. (2010)]. In turn, each specific relationship and corresponding management style induces corresponding differential responses and attitudes in subordinates, including different performance behaviors (Ilies et al., 2007).

Within this Frontiers Research Topic special issue, there are 13 articles that address these very timely phenomena. Based on comprehensive reading of these articles, we suggest that four themes or meta-narratives can be used to organize the research within this special issue of Frontiers in Psychology.

First, we have several authors who present refinements and ideas that consider types of leader-member exchange. Andersen et al. present work that considers the underpinning theoretical perspective of social exchange by presenting descriptions of social-based leader-member exchange and economic-based leader-member exchange as types of sub-constructs. Second, Zhou et al. present the concept of “currencies of exchange” as a way of viewing manifestations of LMX. Here, social currency and work-related currency are the types of exchange constructs that actualize leader-member exchange.

In addition, there are a host of papers, which discuss the role of covariate constructs that play vital roles in how LMX is manifested in workplace environments. Within this issue, constructs as diverse as knowledge sharing behavior (Hao et al., 2019), and various levels of work engagement involving psychological empowerment and psychological withdrawal behavior (Aggarwal et al.), appear as critical behaviors related to leader-member exchange. In respect to individual differences found among employees, this Research Topic includes articles that highlight and add to the literature concerning the critical roles of organizational justice perceptions (Tziner et al., 2012; Fein et al., 2013; Shkoler et al., 2021; Tziner et al.), locus of control (Robert and Vandenberghe), and leader communication styles (Brown and Paz-Aparicio), which have been used to extend the efficacy of leader-member exchange in its association with valued organizational phenomena and outputs.

A third focus of papers within this special issue concerns negative workplace behaviors such as counterproductive work behavior, as well as unethical intentions both from the pro-employee and pro-leader perspectives. Capitalizing on reciprocity theory (Gouldner, 1960), employees in good or bad relationships with their managers (i.e., with high or low LMX) will feel obliged or reluctant to reciprocate mutually to these respective relationships [see also Adams (1965)]. Thus, high- or low-quality LMX results in correspondingly high or low levels of mutual trust, respect, and commitment. Accordingly, subordinates with high LMX relations are likely to receive more rewards (both formal and informal) than their colleagues with lower LMX relations. These benefits include tangible resources, career opportunities, and emotional support (including emotional encouragement), and enhanced feedback (Graen and Uhl-Bien, 1995; Zagenczyk et al., 2015). Consequently, high LMX employees are more likely to engage in more positive behaviors, while those low on LMX will be more prone to negative behaviors (Tziner et al., 2010; Breevaart et al., 2015). Conversely, and in respect to enlarging the network of constructs investigated in this study, it is important to note that poor relations between managers and their employees will almost certainly result in reciprocal counterproductive behavior (Chernyak-Hai and Tziner, 2014). In this issue, counterproductive work behaviors are related to valued organizational outcomes *via* profiles with differing levels of emotional intelligence, as well as cultural value orientations and LMX (Tziner et al.). In respect to negative workplace behaviors, positive and negative reciprocity also occurs as a fundamental construct linked to pro-leader and pro-self-orientations of unethical behavior (Skinner et al., 2018; Vriend et al.) and such forms of reciprocity can also be linked to other global performance dimensions (Fein, 2009).

Finally, there are a number of papers that relate to the focal role of leader-member exchange as a mediating construct. While LMX's role as a potential mediator of workplace misbehaviors has been investigated (e.g., He et al., 2017), most previous studies have emphasized contextual-level or job-level predictors (e.g., He et al., 2017; Sharif and Scandura, 2017). Specifically, we see in this issue that leader-member exchange is critical in linking job insecurity to job satisfaction and turnover intention (Di Stefano et al.), as well as in lowering the tendency of employees to engage in counterproductive work behaviors (Götz et al.; Tziner et al.).

In summary, this issue includes several important contributions to the literature that may be arranged according to these four themes.

## Author Contributions

All authors listed have made a substantial, direct and intellectual contribution to the work, and approved it for publication.

## Conflict of Interest

The authors declare that the research was conducted in the absence of any commercial or financial relationships that could be construed as a potential conflict of interest.

## Publisher's Note

All claims expressed in this article are solely those of the authors and do not necessarily represent those of their affiliated organizations, or those of the publisher, the editors and the reviewers. Any product that may be evaluated in this article, or claim that may be made by its manufacturer, is not guaranteed or endorsed by the publisher.
